# Cardiovascular risk and dyslipidemia among persons living with HIV: a review

**DOI:** 10.1186/s12879-017-2626-z

**Published:** 2017-08-09

**Authors:** Paolo Maggi, Antonio Di Biagio, Stefano Rusconi, Stefania Cicalini, Maurizio D’Abbraccio, Gabriella d’Ettorre, Canio Martinelli, Giuseppe Nunnari, Laura Sighinolfi, Vincenzo Spagnuolo, Nicola Squillace

**Affiliations:** 1 Clinica Malattie Infettive Policlinico, Bari, Italy; 2 Clinica Malattie Infettive, Policlinico Ospedale S. Martino, Genoa, Italy; 3 Divisione Clinicizzata di Malattie Infettive, DIBIC L. Sacco, Università degli Studi di Milano, Milan, Italy; 4 INMI L. Spallanzani, Rome, Italy; 5 UOC. di Immunodeficienze e Malattie Infettive di Genere, P.O. “D. Cotugno”, AORN Dei Colli, Naples, Italy; 6 Clinica Malattie Infettive. Policlinico Umberto I, Rome, Italy; 7 SOD Malattie Infettive e Tropicali AOU Careggi, Florence, Italy; 8 Clinica Malattie Infettive Policlinico, Messina, Italy; 9 Malattie Infettive AO, Ferrara, Italy; 10 Dipartimento Malattie Infettive Ospedale S. Raffaele, Milan, Italy; 11 Divisione Malattie Infettive AO S. Gerardo, Monza, Italy

**Keywords:** HIV, Cardiovascular risk, Statins, Ezetimibe, Fibrates, Omega 3 fatty acids ART, Lipodystrophy, Dyslipidemia

## Abstract

**Background:**

Aim of this review is to focus the attention on people living with HIV infection at risk of developing a cardiovascular event. What is or what would be the most suitable antiretroviral therapy? Which statin or fibrate to reduce the risk? How to influence behavior and lifestyles?

**Discussion:**

Prevention of cardiovascular disease (CVD) risk remains the first and essential step in a medical intervention on these patients. The lifestyle modification, including smoking cessation, increased physical activity, weight reduction, and the education on healthy dietary practices are the main instruments.

Statins are the cornerstone for the treatment of hypercholesterolemia. They have been shown to slow the progression or promote regression of coronary plaque, and could also exert an anti-inflammatory and immunomodulatory effect. However the current guidelines for the use of these drugs in general population are dissimilar, with important differences between American and European ones. The debate between American and European guidelines is still open and, also considering the independent risk factor represented by HIV, specific guidelines are warranted.

Ezetimibe reduces the intestinal absorption of cholesterol. It is effective alone or in combination with rosuvastatin. It does not modify plasmatic concentrations of antiretrovirals. A number of experimental new classes of drugs for the treatment of hypercholesterolemia are being studied.

Fibrates represent the first choice for treatment of hypertriglyceridemia, however, the renal toxicity of fibrates and statins should be considered.

Omega 3 fatty acids have a good safety profile, but their efficacy is limited. Another concern is the high dose needed. Other drugs are acipimox and tesamorelin.

Current antiretroviral therapies are less toxic and more effective than regimens used in the early years. Lipodistrophy and dyslipidemia are the main causes of long-term toxicities. Not all antiretrovirals have similar toxicities. Protease Inhibitors may cause dyslipidemia and lipodystrophy, while integrase inhibitors have a minimal impact on lipids profile, and no evidence of lipodystrophy. There is still much to be written with the introduction of new drugs in clinical practice.

**Conclusions:**

Cardiovascular risk among HIV infected patients, interventions on behavior and lifestyles, use of drugs to reduce the risk, and switch in antiretroviral therapy, remain nowadays major issues in the management of HIV-infected patients.

## Background

In the recent years we observed an improvement of survival and quality of life in people living with HIV (PLWHIV), due to the success of combined antiretroviral treatment (cART) [1].

The early treatment, the reduced toxicity of antiretroviral regimens and the fading of thymidine-analogues-based regimens and the high dosage of ritonavir represent less atherogenic antiretroviral agents for most PLWHIV. This is not enough since PLWHIV live longer, thus in addition to age they add up all degenerative diseases caused by HIV and the side effects of antiretroviral drugs.

This should encourage physicians and researchers in seeking the patient’s well-being, not only through HIV-RNA suppression, but thinking about other more ambitious goals, perhaps more distant from infectious diseases.

Aim of this review is to focus the attention on PLWHIV at risk of developing a cardiovascular event. What is the most suitable cART? Which statin or fibrate to use in order to reduce the risk? How to influence behavior and lifestyles? Everything in the coming years will be played in this field, so we must be prepared.

### Prevention of cardiovascular events

An inappropriate lifestyle, in particular smoking, reduced exercise, unhealthy diet and psychosocial stress are responsible for an increased CVD risk. The “*lifestyle*” is generally based on established patterns of behaviour over time, which have been internalized from childhood and adolescence through the interaction of genetic and environmental factors and that are maintained or even encouraged by the social context in adulthood age.

The dietary habits and physical activity in particular are key factors for the reduction of CV diseases: risk factors such as alcohol use, high blood pressure, high body mass index, hypercholesterolemia, diabetes, low fruit and vegetable intake and physical inactivity, collectively account, with smoking, for more than 60% of cardiovascular deaths globally [2]. Energy intake should be limited to the amount of energy needed to maintain (or obtain) a healthy weight, that is a BMI >20.0 but <25.0 kg/m^2^.

The wide variety of foods of animal and vegetable origin is the basis for a healthy and balanced diet. Many published data show that the Mediterranean diet appears to be protective against cardiovascular disease and total mortality. The use of this type of diet can have beneficial effects not only on prevention of the main CVD risk factors but also on the course of the disease once it presented. The recommendations of the Mediterranean diet are reasumed in Table 1.

**Table 1:** Main recommendations of the Mediterranean diet [127–131]

Increase the consumption of fresh fruit and vegetables of all kinds
Increase the consumption of legumes such as beans, peas, chickpeas and lentils
Eat fish two or three times a week
Encourage the use of extra-virgin olive oil and sunflower and maize oils
Limit the consumption of animal saturated fat acids such as butter, lard and cream

Smoking remains the main responsible for the majority of CVD [3]. The 10-year fatal CVD risk is approximately doubled in smokers. The RR in smokers <50 years of age is five-fold higher than in non-smokers [4].

Preventing PLWHIV from starting smoking is critical, because stop smoking remains a formidable challenge. Although the percentage of smokers is declining in Europe, it still remains high and is rising in women, including adolescents and people socially disadvantaged [5]. Widening education-related inequalities in smoking cessation rates have been observed in many European countries. The risks associated with smoking show a dose-response relationship with no lower limit for deleterious effects [4]. Duration also plays a role, and while cigarette smoking is the most common, all types of smoked tobacco, including low-tar (‘mild’ or ‘light’) cigarettes, filtered cigarettes, cigars and pipes, are harmful [6]. There is no age limit to the benefits of smoking cessation.

Some studies have investigated how much the increased mortality in PLWHIV was attributable to smoking. In 2013, a Danish study [7] observed that the cohort of PLWHIV who smoked had an increased risk of death from causes non-AIDS related at least four times higher. A more recent analysis of the same cohort [8] shows that among HIV people who have never smoked the risk of heart attack is similar to that of HIV-uninfected subjects; in contrast, among the HIV active smokers the risk was three times larger than that observed in HIV negative smokers. The Danish data can be reproduced in the HIV population of Western Europe who started an antiretroviral treatment. Researchers in the ART Cohort Collaboration [9] analyzed all living patients in antiretroviral therapy by at least one year, for whom data on smoking habits were available between January 1999 and December 2008. However, they excluded the intravenous drug users because in these subjects the habit of smoking is widespread and mortality rates are higher than the rest of the HIV population. The analysis took into account 17,995 individuals for a total follow-up of 79,760 person-years. Of these 60% were smokers. According to the results, the mortality rate due to all causes was 7.9 (CI 95%: 7.2 to 8.79) per 1000 person-years among smokers and 4.2 (CI 95%: 3.5 to 5.0) among non-smokers.

Finally, it as to be remembered that some ways in which people cope with stress such as drinking, smoking or overeating, are not healthy especially in PLWHIV.

## Combination antiretroviral therapy (cART) switching

### General principles

Switching from current cART to a more lipid-friendly regimen may represent an option to improve dyslipidemia and to reduce cardiovascular risk in PLWHIV. The principles of cART switching also in the setting of dyslipidemia are to maintain virologic suppression, improve adherence and tolerability [10, 11]. Regimen or single drug substitution may be done carefully and based on the review of different patterns: a) individual factors contributing to dyslipidemia; b) complete clinical and cART regimens history; c) virologic responses to previous cART regimens; d) historical genotypic resistance test; e) adherence history; f) previous cART associated toxicities. Besides, influence of current cART regimen on lipids profile could be evaluated. Clinical trial data on naïve patients demonstrated that differences antiretroviral classes influence lipid values, even if there is heterogeneity among agents within every class: ritonavir (RTV) has been shown to significantly increase plasma lipid levels, in particular lopinavir/ritonavir (LPV/r) and fosamprenavir/ritonavir (FPV/r), to lesser extent with atazanavir/ritonavir (ATV/r) [12, 13].

At the CROI 2017, much attention was centered on the D.A.D, findings on darunavir (DRV) in regards to the development of cardiovascular events. Their analysis had been based on 7 years of follow-up and linked the cumulative use of DRV/r to a gradually increasing risk of CVD [14]. CVD incidence with cumulative DRV was similar to that seen with old-times PIs indinavir and LPV/r. Authors did not show any clear explanation yet, after indicating that abnormal lipids did not modify the CVD risk with DRV/r, and did not provide any distinction between subjects treated with DRV/r BID versus QD.

In the class of nucleoside/nucleotide reverse transcriptase inhibitors (NRTIs), dyslipidemia has been associated with exposure to stavudine, zidovudine and abacavir, whereas tenofovir disoproxil fumarate (TDF) has been noted to have a favourable lipid influence [15, 16]. In the NNRTIs class, efavirenz has been associated with increases in total cholesterol and triglycerides, whereas rilpivirine has been shown to have less effect on lipid parameters than efavirenz [17]. Data on etravirine (ETR) are limited [18]. The integrase inhibitors (INIs) have a little effect on lipid profile [19–21], as well as C-C chemokine receptor type 5 (CCR5) inhibitor (maraviroc) [22]. Since each cART switching has a potential virologic failure risk, it is recommended to prefer regimens that are supported by clinical trials, switch studies or observational cohort studies. The potential options to appropriately switch cART agents are listed below:

Switching within NRTIs: there is evidence of improvement of total cholesterol (TC), LDL-C and TGL switching from abacavir (ABC)/lamivudine to TDF/emtricitabine (FTC) [23, 24].

Switching from protease inhibitor boosted (PI/r) to NNRTIs: Most data on effect of switching from PI/r to nevirapine or efavirenz derived from the early cART era [25, 26]. In the recent years, the randomized SPIRIT study, switching from PI/r to rilpivirine (RPV) has been associated with improved lipid parameters and 10-year Framingham score [27]. In the ETRASWITCH study the switch from PI/b to ETR showed a significant reduction in total cholesterol, TGL and glycemia [28].

Switching from PI unboosted to NNRTI: in a most recent observational trials switch from unboosted PI to RPV has been associated with significant improved of lipids parameters [29].

Switching from PI/r to an INSTI: Switching studies from PI/r to raltegravir (RAL) (SWITCHMRK and SPIRAL) demonstrated a significant lipid and cardiovascular biomarkers reduction in RAL arm. However, in the SWITCHMRK study it was noticed an increased risk of virologic failure that was probably linked to a bias in the patient’s selection [30, 31]. In the open-label STRATEGY study, including nearly 433 participants on first- or second-line treatment regimens with no previous virologic failure, were randomly assigned (2:1) to switch to elvitegravir/cobicistat (EVG/COBI)/FTC/TDF or continue stable PI/r regimens. At week 48, gastrointestinal symptoms improved in the group switched to EVG/COBI/FTC/TDF. Moreover, the INI based regimens led to significant decreases in total cholesterol, triglycerides and HDL-c in LPV/r switches, decrease in triglycerides in ATV/r switches and increase in HDL-c in darunavir switches [32].

### Switch strategies in antiretroviral therapy in the management of dyslipidemia

Antiretroviral therapy suppresses viral replication and reduces the concentration of systemic immune activation markers [33, 34] and several studies reported the influence of therapy modification on biomarkers of CVD (Table 2). On the other hand, multiple evidences suggest that some antiretrovirals such as the class of PI and the NRTI abacavir can contribute to the increased cardiovascular risk observed among HIV infected subjects [35]. Protease inhibitors are associated to higher rates of dyslipidemia and increase of intima-media thickness [12]. ABC has been initially reported to increase the risk of myocardial infarction by data coming from the D:A:D in 2008 [36]. The supposed mechanism is that this drug can be associated to endothelial disfunction and/or increased platelets activation. Subsequent reports from the D:A:D study highlighted that abacavir might represent a risk factor especially for patients presenting other preexisting cardiovascular risk factors, but further analyses from the Food and Drugs Administration did not link ABC to increased frequencies of cardiovascular episodes, thus did not lead to restrictions in the prescription and use of ABC [37]. Several trials have shown a beneficial effect of switching to a NNRTI-containing antiretroviral regimen, as it is shown in Table 3.

**Table 2:** Switch studies reporting the influence of therapy modification on biomarkers of cardiovascular disease

Study Acronym	Study Design	Enrolled patients	Markers evaluated	time endpoint evauation	Laboratory markers change	reference
STRATEGY- NNRTI	Randomized, open label switch study looking at the non inferiority of switching patients who were virologically suppressed on an NNRTI based regimen to co-formulated elvitegravir	439 patients. 266 out of 291 participants randomized to the switch group completed the study. 119 out of 143 participants assigned to the no-switch group completed the study	Fasting serum cholesterol; fasting serum HDL-C; fasting serum LDL-C; fasting serum triglyceride; CD4 cell count; HIV RNA;	48	At week 48 93% of participants in the switch group and 88% in the no-switch group maintained plasma viral load <50 copies/ml. No emergent resistances were observed among the two groups. Starting at week 4 increases of serum creatinine were observed among the switch group; increase was stable and non progressive through week 48. A small decrease in HDL-C was observed in the switch group.	[32]
STRATEGY PI	Multicenter randomised open-label trial investigating the non inferiority of switching to co-formuated elvitegravir in patients virologically suppressed on a PI based regimen	433 patients. 293 were included in the group switching to co-formulated elvitegravir and 140 remained on their existing regimen	Fasting serum COL; fasting serum HDL-C; fasting serum LDL-C; fasting serum TG; CD4 cell count; HIV RNA;	48	At week 48 3.8% of patients enrolled in the switch arm abd 87.1% of participants in the no-switch arm maintained a plasma HIV RNA <50 copies/ml. Starting at week 4 a stable non progressive increase in serum creatinine occurred among switch arm participants. A decrease in serum triglycerides was observed in the switch group.	[32]
SPIRAL Substudy	Changes in cardiovascular biomarkers in HIV-infectedpatients switching from ritonavir-boosted proteaseinhibitors to raltegravir	Of 273 patients initiating study drugs in the SPIRAL trial, 233 (119 RAL, 114 PI/r) remained on allocated therapy for 48 weeks and had sera available for the purpose of this substudy	hsCRP MCP-1 OPG IL-6 IL-10 TNF-a ICAM-1 VCAM-1 Selectin E Selectin P Adiponectin Insulin D-dimer	48	hsCRP (40%, *P* < 0.0001), MCP-1 (20%, P¼0.0003), osteoprotegerin (13%, P¼0.0024), IL-6 (46%,*P* < 0.0001), TNF-a (27%, P¼0.0011), insulin (26%, *P* < 0.0001), and D-dimer (8%, P¼0.0187) decreased in RAL relative to PI/r group, whereas IL-10 (þ1%, P¼0.7773), ICAM-1 (6%, P¼0.1255), VCAM-1(0%, P¼0.8671), E-selectin (9%, P¼0.2174), P-selectin (6%, P¼0.3865), and adiponectin (þ8%, P¼0.2028) remained unchanged	[34]
SPIRAL Substudy LDL	LDL subclasses and lipoprotein-phospholipase A2 activity in suppressed HIV-infected patients switching to raltegravir	81 (41 PI/r and 40 raltegravir) patients were evaluated	Total cholesterol, LDL-c, HDL-c,Triglycerides, TC/HDL-c,Non-HDL-c, Apo A-I, Apo B, ApoA-I/Apo B Lipoprotein PCSK9,LDL size, Cholesterol content in sdLDLLDL phenotype A, LDL phenotype intermediate, LDL phenotype B, Lp-PLA2 Total LDL-Lp-PLA2 Total HDL-Lp-PLA2 8 (4; 14.9) 8.7 (5.4; 17.2) 0.829Insulin, C-Peptide, HOMA index	48	TC, LDL-c, non-HDL-c, TC/HDL, triglyceride, Apo B, Apo A-I and Lp (a) decreased in raltegravir arm compared to PI/r arm. A shift from LDL phenotype B to the less atherogenic phenotype A was observed only in raltegravir arm.	[31]
SPIRAL-LIP substudy	To compare 48-week changes in body fat distribution and bone mineral density (BMD) - using Dual-energy X-ray absorptiometry and computed tomography scans - between patients switching from a ritonavir-boosted protease inhibitor (PI/r) to raltegravir (RAL) and patients continuing with PI/r.	86 patients were included and 74 patients (39 RAL, 35 PI/r) completed the substudy.	**CT-scan:**TAT (cm2) SAT (cm2) SAT (%) VAT (cm2) VAT (%)SAT/VATSAT, subcutaneous adipose tissue; TAT, total adipose tissue; VAT, visceral adipose tissue	48	Significant increases in median VAT and TAT were seen within the PI/r group.No significant changes in body fat were seen with RAL or between treatment groups.	[34]
ANRS 138 Substudy	To compare the effect of randomly switching virologically suppressed, treatment-experienced patient from enfuvirtide to raltegravir on biomarker levels	164 participants in the ANRS138 trial	IL-6 hsCRP Level D-dimer	24, 48	At week 24, a significant decrease from baseline was observed in the IS arm, compared with the DS arm, for IL-6 level (−30% vs +10%; *P* < .002), hsCRP level (−46% vs +15%; *P* < .0001), and D-dimer level (−40% vs +6%; *P* < .0001). At week 48, there was a reproducible decrease in levels of all biomarkers in the DS arm	[132]
na	We retrospectively identified from our electronic database all patients with HIV RNA < 50 copies/ml for >6 months on an NVP-containing regimen and no prior exposure to integrase strand transfer inhibitors who were switched to RAL plus NVP.	39 patients	Total cholesterol, HDL-cholesterol,LDL-cholesterol,Total cholesterol/HDL ratio,triglycerides	24, 48, 72	Median changes in serum lipids showed significant improvement at M6 for all paremeters except low-density lipoprotein-cholesterol in the whole population but lipid improvement was greater in the PI/r group	[133]
na	multi-centric retrospective study was conducted including HIV-1-positive patients on raltegravir/nevirapinedual regimens	77 patients switching from successful regimens	routine biochemical tests	48,96	In patients switching with lipid abnormalities [*n* = 52; 19 (36.5%) on statins, 3 on fibrates (5.7%), 4 on omega-3 fatty acids (7.7%)] triglycerides showed a significant decrease both at 48 and 96 weeks (−83 mg/dL with *p* = 0.004 and −51 mg/dL with *p* = 0.011, respectively), while total and LDL-cl were unchanged (*p* = 0.11 and *p* = 0.12, respectively).	[134]
STRIIVING	randomized open label,non inferiority trial	551 patients on aintegrase inhibitors pr protease inhibitor or nonucleoside reverse trascriptase inhibitor regimens with HIV-RNA < 50 copie/mL were included	TC, HDL-C, LDL C, TG,TC/ratio; hs-PCR,sCD1s,sCD163, IL-6, D-dimer, sVCAM, I-FABP	24	Significant declines of the levels of I-FABP and sCD14 was observed at 24 weeks	[135]

**Table 3:** Trials on the therapeutic switch to a NNRTI-containing regimen

Study Acronym	Study Design and Enrolled Patients	Markers evaluated	time endpoint evauation	Laboratory markers change	Ref
LIPNEFA (a subset of NEFA)	A subset (n. 90) of virologically suppressed HIV-infected patients enrolled in the NEFA study [98] enrolled in the NEFA study were analyzed for the HDL-C and LDL-C levels in the metabolic and body-composition substudy LIPNEFA .	HDL-C and LDL-C levels	24	At 24 months, efavirenz (EFV) and nevirapine (NVP) produced similar lipid benefits: HDL-C levels increased [EFV, 15% (*p* = 0.001); NVP, 21% (*p* < 0.001)] and TC to HDL-C ratios decreased [EFV, 14% (*p* < 0.001); NVP, 19% (*p* < 0.01)].	[25]
SPIRIT	Virologically suppressed HIV-infected adults who were receiving a PI-based HAART were switched to emtricitabine-rilpivirine-tenofovir difumarate (FTC-RPV-TDF).	TC, LDL-C, TGL, and TC:HDL-C ratio.	24	At 24 weeks, levels of TC, LDL-C, TGL, and the TC:HDL-C ratio had improved significantly in patients switched to FTC-RPV-TDF compared to those patients who kept their original PI-based treatment (*p* < 0.001). Nevertheless, HDL-C levels declined significantly less with the PI-based regimen (*p* < 0.001).	[17]
Study 111	Virologically suppressed patients who were switched from EFV-TDF- FTC to RPV-TDF-FTC.	Fasting TC, LDL-C, HDL-C, TGL, and TC:HDL-C ratio.	12, 48	A significative decline in fasting TC, LDL-C and TGL at 12 weeks into the protocol. Results at week 48 remained in the same direction (*p* = 0.016), although changes from baseline in HDL-C and the TC:HDL-C ratio were not significant. The introduction of RPV in the clinical arena has been very positive for the patients care, but we all have to remember the risk of a prolongation of QT interval with higher doses of this compound (i.e. 75 and 300 mg QD) as seen in the early phases of clinical development.	[136]
Etraswitch	The switch from a PI-containing regimen to etravirine (ETR) in patients with therapeutic success.	Glycemia, fasting TC, LDL-C, HDL-C, TGL, and TC:HDL-C ratio.	24	The group of patients who received ETR showed a significant reduction in TC (*p* < 0.001), TGL (*p* < 0.001), and glycemia (*p* = 0.03). The 2 groups differed significantly in TGL and glycemia at week 24. The greatest improvement in all lipids was seen in patients who switched from lopinavir/ritonavir to ETR.	[28]
na	A prospective, open-label, 12-week study of HIV-infected patients receiving either a on bPI or EFV, and statin treatment. Four weeks after statin interruption, bPI or EFV was switched to ETR (400 mg for 8 weeks) if serum low-density lipoprotein cholesterol (LDL-C) was ≥3 mM. The primary endpoint was the proportion of patients not qualifying for statin treatment 8 weeks after the ETR switch.	Fasting TC, LDL-C, HDL-C, TGL, and TC:HDL-C ratio.	12	After 8 weeks of ETR treatment, 15 patients (56%) on ETR did not qualify for statin treatment. After the ETR switch, serum levels of the cardiovascular biomarkers sICAM and MCP1/CCL2 decreased by 11.2% and 18.9%, respectively, and those of CCL5/RANTES and tissue inhibitor of metalloproteinase-1 increased by 14.3% and 13.4%, respectively, indicating reduced cardiovascular risk.	[137]

### Monitoring after switching cART regimen

During the first 3 months after the switch, the patient should be closely monitored to assess tolerability and adherence to new cART regimen.

HIV-RNA test may be performed to check for rebound viremia 4 weeks after the switch and then every 3 months during the first year; fasting lipids (cholesterol and triglycerides) should be assessed within 3 months after the change and every 3 months during the first year. In absence of new complaints, subsequent monitoring should be performed on a regularly scheduled basis [10, 11].

### New antiretroviral drugs and cardiovascular risk

#### Tenofovir Alefenamide

Tenofovir alafenamide (TAF) is a novel prodrug of tenofovir disiproxil fumarate (TDF). In this new formulations TAF show higher levels of TDF-DP in periphereal blood mononuclear cells and lymphoid tissues than TDF. TAF showed safety advantages compared to TDF containing regimen regarding bone and renal toxicity [38], regarding the lipid profile, increase from baseline in fasting total cholesterol, LDL cholesterol, HDL and triglycerides were observed in TAF than with TDF. TAF has no “statin-like” phenomenon which characterized TDF. Long-term data are not know.

#### Cabotegravir

Cabotegravir is an investigational HIV INI drug, currently being studied in Phase IIb clinical trials. During preliminary studies conducted on healthy subjects both in monotherapy and in combination with RPV [39, 40] no consistent, clinically significant, or dose-related changes in haematology, clinical chemistry, vital signs, or ECG abnormalities or trends were observed. Lou et al. [41], in a study that assessed its effect on cardiac repolarization in healthy subjects, demonstrated that cabotegravir at a supratherapeutic dose had no effect on cardiac repolarization. In a fase IIa study, Spreen et al. [42] described two cases of laboratory abnormalities in HIV-1 infected patients in Cabotegravir monotherapy arm. A subject with type I diabetes had grade 2 hyperglycemia at baseline and at all time points other than day 7 (hypoglycemia) and the follow-up visit (grade 3 hyperglycemia). The other subject, on day 7, had grade 4 TGL elevation subsequent to a very high–fat meal the previous evening, and TGL were within normal limits at all other readings. Even the phase IIb studies, LATTE and LATTE-2 (still ongoing), underlined the good safety profile of cabotegravir with no cases of ECG abnormalities or metabolic disorders [43].

#### Doravirine

Doravirine is a NNRTI currently being studied in Phase III clinical trials as both a single-drug tablet and as part of a fixed-dose combination tablet. In a preliminary study with single and multiple doses of doravirine in healthy subjects, no serious adverse events were reported, and there were no consistent, clinically relevant, treatment-related effects of doravirine on vital signs or ECGs. Overall, no clinically significant trends or signals were observed in laboratory assessments, vital signs or ECGs [44]. In a randomized, double-blind, placebo-controlled, short-term monotherapy study of doravirine Shurmann et al. described no clinically significant abnormalities in vital signs, routine blood and urine chemistry panels, haematology, ECGs, or physical or neurological examinations in any participant [45]. Results of a phase IIb clinical trial, presented by Gatell et al. at the 12th International Congress on HIV Drug Therapy being held in Glasgow, showed that 6.8% of patients had an increase in total cholesterol and 6.3% an increase of LDL cholesterol [46].

## Pharmacological treatment of dyslipidemia

### Drugs for the treatment of hypercholesterolemia

#### Statins

Statins are the cornerstone of the treatment of hypercholesterolemia. They reduce the synthesis of cholesterol in the liver by competitively inhibiting the hydroxy-3-methylglutaryl coenzyme A (HMG-CoA) reductase activity. The reduction in intracellular cholesterol concentrations induces LDL receptor expression on the hepatocyte cell surface, which results in increased extraction of LDL-cholesterol (LDL-C) from the blood and a decreased concentration of circulating LDL-C and other lipoproteins including triglycerides-rich particles.

A number of large-scale clinical trials have demonstrated that statins substantially reduce cardiovascular (CV) morbidity and mortality in both primary and secondary prevention [47, 48]. In the general population, each 1.0 mmol/L (39 mg/dl) reduction in LDL-C level has been significantly associated with a 10% reduction in all-cause mortality, largely reflecting significant reductions in deaths due to coronary heart disease [47].

Meta-analyses of randomized controlled trials that focused on primary prevention, demonstrated that in people without established CV disease but with CV risk factors, the use of statins was associated with significantly improved survival and large reductions in the risk of major CV events [48, 49]. Benefits of statin therapy were observed in nearly all subgroups, including persons with diabetes mellitus, men and women, and across age groups.

The most recent clinical trials suggested that the LDL-C lowering effect of statins between PLWHIV is similar to that seen in the general population. Several randomized studies of hypercholesterolemic HIV-infected patients showed that LDL-C decreased by 15% to 35% in patients taking statins as compared with placebo [50]. In a trial in which PLWHIV on protease inhibitors (PIs) were randomized to atorvastatin 10 mg, rosuvastatin 10 mg, or pravastatin 20 mg, the mean reductions in the LDL-C at one year were 20%, 25%, and 18%, respectively, after one year of therapy [51].

Recently, statins have also been shown to slow the progression or even promote regression of coronary atherosclerosis in the general population as well as in HIV-infected patients (Table 4).

**Table 4:** Effects of statin therapy on the progression of atherosclerosis in HIV- and non-HIV-infected patients

Type of study	Method	N°of patients	Effect of statin therapy	Ref
Meta-analysis	Coronary VH-IVUS	830 non HIV-infected patients	Reduction of plaque volume	[138]
Randomized double-blind, placebo controlled trial	FDG-PET of the aorta	40 HIV-infected patients	Reduction of plaque volume and high-risk plaque morphology over 52 weeks	[139]
Randomized double-blind, placebo controlled trial	Common carotid artery IMT	147 HIV-infected patients on stable ART	Less progression of CCA IMT over 96 weeks	[52]

VH-IVUS, virtual histology intravascular ultrasound; FDG-PET, Fluorodeoxyglucose-positron emission tomography; IMT, intima media thickness; ART, antiretroviral therapy; CCA, common carotid artery

Finally, as rosuvastatin has demonstrated to significantly reduce several markers of vascular inflammation and CD4+ and CD8+ T lymphocyte and monocyte activation in HIV-infected subjects on antiretroviral therapy [52, 53], statins could exert a wide-reaching anti-inflammatory and immunomodulatory effect that extends well beyond CV disease prevention.

Although the effects of statins in HIV-infected patients are expected to be of a similar magnitude to that has been seen in the general population, many issues regarding the use of statins in HIV-infected patients are still unclear, and further studies are needed to determine whether statins reduce the number of CV events and mortality in HIV-infected patients.

The AIDS Clinical Trial network is currently enrolling in the REPRIEVE (Randomized Trial to Prevent Vascular Event in HIV) Study, which is a large-scale randomized trial to investigate daily pitavastatin versus placebo for the primary prevention of CV events in PLWHIV. Until the results of such studies specifically conducted in HIV-infected population will be available, we have to relay on guidelines for non-HIV-infected patients for the management of hypercholesterolemia in persons who are living with HIV.

For the use of statins in CV disease (CVD) prevention, the European Society of Cardiology and the European Atherosclerosis Society Guidelines, suggest to evaluate the total CV risk of the subjects using European SCORE tables and to identify the LDL-C target for that risk level. For these guidelines, since the response to statin treatment is variable, up-titration to reach that target is mandatory [54].

Differently from European guidelines, the 2013 American College of Cardiology and the American Heart Association guidelines (ACC/AHA) guidelines introduced a new risk calculator, and identified four groups of patients who should be treated with a statin (Table 5) [55]. Current available evidence suggests that the clinical benefit is largely independent of the type of statin but depends on the extent of LDL-C lowering; therefore, the type of statin used should reflect the degree of LDL-C reduction that is required to reach the target LDL-C in a given patient [56].

**Table 5:** The 2013 ACC/AHA guideline statin benefit group

1) Patients **with clinical atherosclerotic CVD** ^a^	
2) Patients without clinical atherosclerotic CVD, **with LDL-C level ≥ 190 mg/dl**	
3) Patients aged 40–75 years, **with type I or II diabetes mellitus**, and LDL-C level < 190 mg/dl	
4) Patients without clinical atherosclerotic CVD or diabetes, aged 40–75 years, with LDL-C < 190 mg/dl, and **an estimated 10-year CV risk ≥ 7.5%** ^b^	

CVD, cardiovascular disease; CV, cardiovascular

^a^including acute coronary syndrome, myocardial infarction, angina, revascularization, transient ischemic attack, stroke, peripheral arterial disease

^b^calculated using the 2013 ACC/AHA risk assessment tool

At maximal recommended dose, the different statins differ in their LDL-C lowering capacity. The ACC/AHA guidelines classify statin doses by three levels of intensity, based on their ability to lower LDL-C levels in the general population (Table 6) [55]. On the other hand, this Expert Panel did not find evidence to support titrating statin therapy to achieve optimal LDL-C or non- high-density lipoprotein cholesterol (HDL–C) targets.

**Table 6:** The ACC/AHA guideline statin dose classification

	Reduction of LDL-C level			
		**50%**	**30–50%**	**≤30%**
**Intensity dose (dose of statin daily)**				
	**High**	Atorvastatin 20–40 mg Rosuvastatin 20–40 mg		
	**Moderate**		Atorvastatin 10–20 mg Rosuvastatin 5–10 mg Simvastatin 20–40 mg Pravastatin 40–80 mg Fluvastatin 80 mg Pitavastatin 2–4 mg	
	**Low**			Simvastatin 10 mg Pravastatin 10–20 mg Lovastatin 20 mg Fluvastatin 20–40 mg Pitavastatin 1 mg

LDL-C, low-density lipoprotein-cholesterol

Other considerations when deciding about statin therapy include: diabetes mellitus in individual aged less than 40 years or more than 75 years; a family history of premature CVD; elevated lifetime risk of CVD; LDL-C levels of 160 mg/dl or higher; a high sensitivity C-reactive protein level of 2.0 mg/L or higher; a coronary artery calcium score of 300 or higher; an ankle-brachial index below 0.9 [55].

In addition, as HIV-infected patients have been shown to have an increased risk for CVD compared with the general population [57], the presence of HIV infection per se should be considered as an additional risk factor.

The main objection to the ACC/AHA guidelines is that, if generally adopted, these would result in an increased number of patients treated, potentially at considerable costs. Moreover, the new pooled mixed cohorts equation used to assess atherosclerotic CVD risk, has been validated in an American population, different from European countries and requires more careful evaluation if applied in other contexts [58]. In summary, the debate between American and European guidelines is still open and considering the independent risk factor represented by HIV, specific guidelines for HIV-infected persons are warranted.

Statins are generally well tolerated, and serious adverse events are rare [59]. The most serious adverse effects associated with statin therapy is myopathy, which may progress to rhabdomyolisis, and that, in turn, can lead to a concomitantly increased risk of renal failure [59]. An elevation of creatine phosphokinase (CK) is the best indicator of statin-induced myopathy. The common definition of tolerable elevation is a rise of four times the upper limit of normal (ULN) of this enzyme measured in two occasions [59]. The incidence of myopathy is low (<1/1000 patients treated) and the excess risk in comparison with placebo-treated patients has been <1/10,000 patients treated in clinical trials. The incidence of rhabdomyolisis associated with statin therapy is about 1/100.000 per year. Myopathy is most likely to occur in persons with complex medical problems and/or who are taking multiple medications, or in elderly persons, especially women. Myalgia without CK elevation occurs in 5–10% of patients in clinical practice [59].

Combination of statins with fibrates may enhance the risk of myopathy. This risk is greater for gemfibrozil, and the association of gemfibrozil with statins should be avoided. The increased risk for myopathy, when combined statins and fibrates seems to be small [60]. The increased risk for myopathy with nicotinic acid has been debated, but in recent reviews no increased risk of myopathy was found with this agent [61].

The increased risk of diabetes with statin is unclear and high-dose statin are more likely to be associated with diabetes than lower doses [62]. In a randomized, placebo-controlled trial of rosuvastatin versus placebo in antiretroviral treated HIV-infected patients, statin therapy showed a more than 50% increase in insulin resistance, as measured by the homeostasis model assessment of insulin resistance (HOMA-IR), compared with placebo. Nevertheless, there were no increase in the incidence of diabetes and no significant change in oral glucose tolerance testing [63]. The main concern about the use of statins in HIV-infected patients is represented by their potential interactions with some antiretrovirals that may increase the risk of side effects. All current available statins, except pravastatin, rosuvastatin, and pitavastatin, undergo major hepatic metabolism through the cytochrome P (CYP) system thus, other pharmacological substrates of these CYPs may interfere with statin metabolism. Conversely, statin therapy may interfere with the catabolism of other drugs that are metabolized by the same enzymatic system. PIs and other antiretrovirals, such efavirenz, interact with statins because they potentially inhibit CYP3A4 or transporters or both. The potential interactions with antiretrovirals can be managed with careful selection of the appropriate statin, often at lower dose than that is used in the general population [9]. Simvastatin has greater toxicity when combined with PI-containing regimens and is contraindicated in PLWHIV on PIs. Pravastatin and rosuvastatin generally are considered the safer statins because their metabolism does not utilize CYP3A4. Pitavastatin appears to have a particularly favourable pharmacokinetic profile and is not known to interact with current available antiretroviral drugs, even in the setting of PI coadministration [64].

#### Non-statin drugs for the treatment of hypercholesterolemia

In patients either unable to achieve optimal LDL-C levels despite statin treatment or intolerant to statin, the availability of new drugs with LDL-C lowering effects may be beneficial for reducing atherosclerotic cardiovascular disease (ASCVD) risk.

#### Ezetimibe

Ezetimibe is a lipid lowering drug that inhibits the intestinal absorption of cholesterol withouth significant interaction with P-450 cytocromes [64].

Studies on PLWHIV with dyslipidemia evaluated ezetimibe alone or in combination with statins. Ezetimibe alone showed a statistically significant reduction of LDL-cholesterol (ranging from 5.3% to 20.4%) but no significant change in HDL-cholesterol and triglycerides [65, 66]. The studies that added ezetimibe to a stable statin therapy showed a significant reduction of total cholesterol (ranging from −12.9% to −21%) and LDL-C (from 20.8% to 35%) with conflicting results about HDL and triglycerides. Ezetimibe represents an affordable choice in statin-intolerant patient and a good option to intensify statin therapy with a very low toxicity profile [67–73].

#### PCSK9 inhibitors

Proprotein convertase subtilisin/kexin type 9 (PCSK9) gene, discovered in 2003, encode for a serine protease protein that plays a central role for the regulation of cholesterol metabolism by targeting the LDL receptor (LDLR) for the degradation in the liver [74]. Gain-of-functions mutations in PCSK9 are one of the genetic causes of autosomal dominant hypercholesterolemia [75]. On the other hand, low-of-function mutations are associated with lower levels of LDL-C and reduced rates of coronary artery disease [76].

Several strategies have been developed to block PCSK9 function including binding of plasma PCSK9 with neutralizing monoclonal antibodies (mAbs) or targeting the intracellular PCSK9 by antisense oligonucleotides or small interfering RNA. Up to date the most studied and clinically advanced approach to PSCK9 inhibition is the use of mAbs.

Two mAbs (alirocumab and evolocumab) have been recently approved by the European Medicines Agency for the use in subjects with familial hypercholesterolemia, in patients who failed to achieve acceptable lipid control although an optimal lipid lowering therapy and in those intolerant to statins.

Efficacy and tolerability of alirocumab and evolocumab in the general population has been evaluated in different studies, as reported in Table 7 [77–85].

**Table 7:** Efficacy and safety of evolocumab and alirocumab in different studies performed in the general population

Study Design	Enrolled patients	Efficacy results	Safety results	reference
Meta-analysis on Phase 2 or 3 randomized, controlled trials (RCTs) comparing treatment using PCSK9 antibodies with no anti-PCSK9 therapy in adults with hypercholesterolemia.	Twenty-four RCTs comprising 10,159 patients were included.	Treatment with PCSK9 antibodies led to marked reductions in LDL-C (mean difference, −47.49% [95% CI, −9.64% to −25.35%]; *P* < 0.001] and it reduced all-cause mortality ([OR], 0.45 [CI, 0.23 to 0.86]; *P* = 0.015; heterogeneity *P* = 0.63; I2 = 0%) and cardiovascular mortality (OR, 0.50 [CI, 0.23 to 1.10]; *P* = 0.084; heterogeneity *P* = 0.78; I2 = 0%).	The overall incidence of serious adverse events was 9.26% (573 of 6187) among patients treated with PCSK9 antibodies and 7.73% (307 of 3972) among patients who were not treated with PCSK9 antibodies (OR, 1.01 [CI, 0.87 to 1.18]; *P* = 0.879; heterogeneity *P* = 0.98; I2 = 0%).	[77]
Meta-analysis to evaluate the safety and efficacy of anti-PCSK9 antibodies in randomized, controlled trials (RCTs).	Twenty-five RCTs encompassing 12,200 patients were included	Evolocumab treatment significantly reduced LDL-C by −54.6% and by absolute −78.9 mg/dl versus placebo, and by −36.3% versus ezetimibe. Alirocumab lowered LDL-C by −52.6% versus placebo, by −29.9% versus ezetimibe.	Alirocumab was associated with an increased rate of injection-site reactions (RR: 1.48, 95% CI: 1.05 to 2.09, *P* = 0.02)	[78]
ODYSSEY FH I and II: two randomized, double-blind studies to assess long-term alirocumab in patients with heterozygous familial hypercholesterolaemia	ODYSSEY FH I, *n* = 486; FH II, *n* = 249 subjects	Mean LDL-C levels decreased from 3.7 mmol/L (144.7 mg/dL) at baseline to 1.8 mmol/L (71.3 mg/dL; 257.9% vs. placebo) at Week 24 in patients randomized to alirocumab in FH I and from 3.5 mmol/L (134.6 mg/dL) to 1.8 mmol/L (67.7 mg/dL; 251.4% vs. placebo) in FH II (P, 0.0001).	Adverse events resulted in discontinuation in 3.4% of alirocumab-treated patients in FH I (vs. 6.1% placebo) and 3.6% (vs. 1.2%) in FH II. Rate of injection site reactions in alirocumab-treated patients was 12.4% in FH I and 11.4% in FH II (vs. 11.0 and 7.4% with placebo).	[80]
TESLA Part B: randomised, double-blind, placebo-controlled phase 3 trial of subjects with homozygous familial hypercholesterolaemia, randomly allocated to receive subcutaneous evolocumab 420 mg or placebo every 4 weeks for 12 weeks.	50 eligible patients	Compared with placebo, evolocumab significantly reduced LDL cholesterol at 12 weeks by 30·9% (95% CI −43·9% to −18·0%; *p* < 0·0001).	Treatment-emergent adverse events occurred in ten (63%) of 16 patients in the placebo group and 12 (36%) of 33 in the evolocumab group.	[81]
ODYSSEY LONG TERM: randomized trial involving subjects to receive alirocumab (150 mg) or placebo for 78 weeks.	2341 patients at high risk for cardiovascular events who had LDL cholesterol levels of 70 mg per deciliter or more and were receiving treatment with statins at the maximum tolerated dose	At week 24, the difference between the alirocumab and placebo groups in the mean percentage change from baseline in calculated LDL cholesterol level was −62% (*P* < 0.001) In a post hoc analysis, the rate of major adverse cardiovascular events was lower with alirocumab than with placebo (1.7% vs. 3.3%; hazard ratio, 0.52; 95% confidence interval, 0.31 to 0.90; nominal *P* = 0.02).	The alirocumab group, as compared with the placebo group, had higher rates of injection-site reactions (5.9% vs. 4.2%), myalgia (5.4% vs. 2.9%), neurocognitive events (1.2% vs. 0.5%), and ophthalmologic events (2.9% vs. 1.9%).	[82]
The GAUSS-2 trial: 12-week, double-blind study of randomized patients (2:2:1:1) to evolocumab 140 mg every two weeks (Q2W) or evolocumab 420 mg once monthly (QM) both with daily oral placebo or subcutaneous placebo Q2W or QM both with daily oral ezetimibe 10 mg.	307 patients	At week 12, evolocumab reduced LDL-C from baseline by 53% to 56%, corresponding to treatment differences versus ezetimibe of 37% to 39% (*p* < 0.001).	Muscle adverse events occurred in 12% of evolocumab-treated patients and 23% of ezetimibe-treated patients. Treatment-emergent adverse events and laboratory abnormalities were comparable across treatment groups.	[83]
OSLER Study: two open-label, randomized trials of patients who had completed 1 of 12 phase 2 or 3 studies of evolocumab.	4465 eligible patients randomly assigned to receive either evolocumab plus standard therapy or standard therapy alone.	As compared with standard therapy alone, evolocumab reduced the level of LDL-C by 61%, from a median of 120 mg per deciliter to 48 mg per deciliter (*P* < 0.001). The rate of cardiovascular events at 1 year was reduced from 2.18% in the standard-therapy group to 0.95% in the evolocumab group (hazard ratio in the evolocumab group, 0.47; 95% CI, 0.28 to 0.78; *P* = 0.003).	Neurocognitive events were reported more frequently in the evolocumab group. The risk of adverse events, including neurocognitive events, did not vary significantly according to the achieved level of LDL cholesterol	[84]
FOURIER study: randomized, double-blind, placebo-controlled trial of patients with atherosclerotic cardiovascular disease and LDL cholesterol levels of 70 mg per deciliter or higher who were receiving statin therapy. The primary efficacy end point was the composite of cardiovascular death, myocardial infarction, stroke, hospitalization for unstable angina, or coronary revascularization	27,564 patients were randomly assigned to receive evolocumab or matching placebo as subcutaneous injections	At 48 weeks, mean percentage reduction in LDL cholesterol levels with evolocumab, as compared with placebo, was 59% (*P* < 0.001). Relative to placebo, evolocumab treatment significantly reduced the risk of the primary end point (1344 patients [9.8%] vs. 1563 patients [11.3%]; hazard ratio, 0.85; 95% confidence interval [CI], 0.79 to 0.92; *P* < 0.001	No significant difference between the study groups with regard to adverse events (including new-onset diabetes and neurocognitive events), with the exception of injection-site reactions, which were more common with evolocumab (2.1% vs. 1.6%).	[85]

Considering the high efficacy of PCSK9 inhibitors in reducing LDL-C levels when used alone or in combination with a statin, their use in HIV-infected subjects may help to control cholesterol levels and probably to reduce the risk of MACE in this patients’ population.

Unfortunately to date, no data on the efficacy of evolocumab and alirocumab among HIV-infected subjects are available. However, a forthcoming randomized trial (NCT02833844) will evaluate safety, tolerability, and efficacy on LDL-C of evolocumab in 450 subjects with HIV and with hyperlipidemia and/or mixed dyslipidemia. Start date is scheduled on May 2017.

#### Drugs for the treatment of hypertriglyceridemia

Isolated hypertriglyceridemia is rare in the setting of PLWHIV on cART in the modern era, the lipid profile usually shows a mixed dyslipidemia [83, 84]. The role of hypertrigliceridemia in cardiovascular disease is still debated [85, 86] and the treatment is reccomended for severe cases (e.g. triglycerides >500 mg/dl) especially for the risk of acute pancreatitis [87].

#### Fibrates

Fibrates represent the first choice for treatment of hypertriglyceridemia in HIV infected patients. They bind and regulate nuclear receptor peroxisome proliferator activator receptor-α (PPAR- α) and regulate gene expression [88]. Fenofibrate is the most commonly used fibrate in HIV-associated dyslipidemia both for the once daily dose and for the reduced interaction with statin with a lower risk of rhabdomyolisis [89, 90]. Studies on HIV-population evaluating fenofibrate showed a significant reduction of triglycerides (from 18% to 58%) depending on cART regimen, study design, and on grade of hypertriglyceridemia [91]. An observational analysis including 80 patients with HIV infection on fenofibrates with a mean baseline triglycerides value of 347 mg/dl showed a reduction of 18% of triglycerides [91]. A randomized trial evaluating fish-oil therapy versus fenofibrate enrolling 50 patients with HIV infection in each arm demonstrated a reduction of 58% of triglycerides in fenofibrate arm with a median baseline of triglycerides of 694 mg/dl [92]. Few studies evaluated the use of fibrates and statins in HIV associated dyslipidemia [93] and they demonstrated an higher efficacy as reported in general population [94]. An important issue is the increased renal toxicity associated with the use of fibrates and statins that should be considered [93].

#### Fish oil

Omega 3 fatty acids have a good safety profile and have been used in HIV-associated dyslipidemia. They demonstrated a reduction of triglycerides ranging from 7% to 38% in a retrospective analysis of 73 patients on PI based regimen vs a non-nucleoside reverse-transcriptase inhibitors (NNRTI) regimen respectively [91]. In three recent randomized trial including less than 50 patients per arm a triglycerides reduction of 9–48% was observed [92–98]. The association with statins results in a more favorable lipid profile with a very low toxicity [99, 100] but the introduction of ezetimibe that shows an higher efficacy has limited the use of omega 3 only for patients with a low-moderate dyslipidemia and isolated hypertrigliceridemia. Another concern is the higher dose (2–4 g/die) needed to obtain a real efficacy with development of side effects such as flatulence.

Studies evaluating fibrates and fish oil in mixed dyslipidemia and isolated trigliceridemia are summarized In Table 8.

**Table 8:** studies evaluating fibrates and fish oil in mixed dyslipidemia and isolated trigliceridemia

Drugs	Sample size	Study Design	Reduction of TRG (±SD or IQR)	References
FO vs Fenofibrate vs Gemfibrozil vs Atorvastatin	76 vs 80 vs 46 vs 291 (HIV-infected).	Observational/ quasi-experimental pre/post design. Median period of observation: 5 months	FO: −45 mg/dl (−80 to −11) Fenofibrates vs FO: −49 mg/dl (−108 to 11) Gemfibrozil vs FO: - 80 mg/dl (−150 to −10) Atorvastatin vs FO: −33 mg/dl (−81 to 15)	[140]
Fenofibrate	55 with Lipodystrophy with severe Hypertriglyceridemia (TRG > 500 mg/dl) [HIV-POS]	Observational/Prospective Observation period = 6 months	Mean change:-335 mg/dl	[90]
FO vs Fenofibrate vs FO + Fenofibrate	50 vs 50, if no response at week 10 switch to combination therapy; 72 pts. switched. (HIV-infected)	Randomized-Open label Study duration: 18 weeks	FO = 46% vs Fenofibrate (58%) vs FO + Fenofibrate (65%)	[95]
Fenofibrate vs pravastatin vs Fenofibrate + pravastatin	88 vs 86, if no response at week 12 switch to combination therapy (most patients switched) [HIV-infected]	Randomized-Open label Study duration: 48 weeks	Median change: −144 mg/dl (−1492 to 927 fenofibrate) vs −66 (−899 to 1567 prevastatin)	[89]
FO	41 (HIV-infected)	Randomized-Open label Study duration: 12 weeks	Mean change: 63.2 ± 86.9 mg/dl mg/dl	[92]
FO	48 (HIV-infected)	Randomized-placebo-controlled Study duration: 8 weeks	Median decrease: −34 (−149–9.5) mg/dL	[97]
FO + simvastatin	254 (uninfected)	Randomized-Open label Study duration: 8 weeks	Mean change: 29% (FO + simvastatin) vs 6% (simvastatin)	[97]
FO + simvastatin	59 with Coronary heart disease already on simvastatin (uninfected)	Randomized, Study duration: 24 weeks + 24 weeks	Mean change: 20–30%	[98]

TRG, Triglycerides; FO, Fish Oil; SD, standard deviation; IQR, interquartile range; pts., patients

#### Tesamorelin

HIV-infected subjects, particularly those treated with antiretroviral therapy, may experience significant accumulation of visceral fat. The increased visceral adiposity has been associated with dyslipidemia and with reductions in growth hormone (GH) secretion [101–103].

The use of tesamorelin, a synthetic analog of human growth hormone-releasing factor, decreases visceral adipose tissue (VAT) and concomitantly reduces tryglicerides, total cholesterol concentration and non-HDL-C among HIV-infected subjects.

In a study by Falutz et al. [104] the measure of VAT decreased by 15.2% in the tesamorelin group and increased by 5.0% in the placebo group; the levels of triglycerides decreased by 50 mg per deciliter and increased by 9 mg per deciliter, respectively, and the ratio of total cholesterol to HDL cholesterol decreased by 0.31 and increased by 0.21, respectively (*P* < 0.001 for all comparisons).

In another study by the same author [105] the use of tesamorelin reduced trygliceride levels by approximately 40 mg/dL in pooled analysis of the ITT population.

Similarly a study by Stanley et al. showed that individuals who responded to tesamorelin (defined as a ≥ 8% reduction in VAT) experienced significantly greater improvements in levels of tryglicerides compared to nonresponders [106].

The observed reduction in tryglicerides is probably mediated at least in part by the direct effect of tesamorelin in increasing GH levels and probably by a possible role of VAT reduction itself in decreasing tryglicerides levels [107].

Tesamorelin is usually well tolerated. The most commonly reported adverse events (>10%) were injection site erythema, pruritus, headache and arthralgia [108].

#### Fat redistribution in HIV-infected subjects

Long-term ART use has been associated with the occurrence of lipodystrophy, a medical condition characterized by an abnormal fat redistribution [109].

Fat redistribution among HIV-infected subjects is usually classified in specific entities: lipoatrophy, lipohypertrophy and mixed syndromes [110, 111].

Lipoatrophy (LA) is the loss of subcutaneous peripheral fat usually at the face, buttocks and limbs [112, 113] and has been associated with the use of thymidine analogues (zidovudine and stavudine). Decline in the use of these drugs resulted in a significant decrease in severe LA prevalence [114].

On the contrary, lipohypertrophy (LH) that is the accumulation of visceral and central fat in the abdomen, anterior neck, dorsocervical region (“buffalo hump”), trunk and/or breasts [115, 116], still occur in some treated patients [117]. In the general population, increased visceral adipose tissue (VAT) increases the risk of type II diabetes mellitus (T2DM), cardiovascular disease (CVD) and overall mortality. In HIV-infected subjects VAT has been also independently associated with mortality [118]. The main hypothesis regarding the development of LH suggests that alterations in peripheral adipocytes result in an increased levels of circulating fatty acid (CFA). The CFA are then deposited in VAT due to the higher rate of lipid turnover and uptake in visceral adipocytes [119]. These alterations in metabolism of adipocyte may be secondary to a direct action of HIV itself via viral protein Vpr or to a deleterious effect of cART [115, 120]. Moreover, ectopic fat deposition is associated with inflammation and adverse metabolic impact beyond that seen with generalized obesity [121]. Associations between intra-abdominal VAT and increased metabolic disease risk (including CVD) are well described both in cross-sectional and longitudinal studies [122–124]. In a cross-sectional study of nearly 600 HIV-infected men on stable ART, greater VAT, liver fat, and epicardial fat were independently associated with CVD after adjusting for traditional CVD risk factors [125]. PLWHIV in the CHARTER study with increased visceral adiposity had significantly worse neurocognitive function. Furthermore, an association between higher IL-6 levels and poorer neurocognitive function was found only among those with the largest waist circumferences, supporting a link between visceral adiposity, inflammation, and neurocognitive function in HIV-infected persons [126].

## Conclusions

Cardiovascular disease and dyslipidemia among PLWHIV remain nowadays challenging issues. Prevention of CVD risk is the first and essential step of medical intervention on these patients. The lifestyle modification and the education on healthy dietary practices are fundamental tools for reducing CVD risk.

Current antiretroviral regimens are easier to take, better tolerated, and more effective than regimens used in the past, therefore optimization of ART in terms of side effects tolerability is essential, since HIV infection requires life-long therapy. The optimal treatment strategies are based on the assumption that not all antiretroviral agents have similar toxicities. PI/ritonavir may cause dyslipidemia and lipodystrophy, while INI have a minimal impact on lipids profile, and not reported evidence of lipodystrophy. There is still much to discover with the introduction of new drugs in clinical practice: dolutegravir, TAF and cobicistat.

Statins are the cornerstone of the treatment of hypercholesterolemia. They slow the progression or even promote regression of coronary atherosclerosis and also could exert a wide-reaching anti-inflammatory and immunomodulatory effect. The 2013 ACC/AHA guidelines introduced a new CV risk calculator different from the ESC and EAS Guidelines for the management of dyslipidaemia and identified 4 groups of patients who should be treated with a statin. These guidelines classify statin doses by 3 levels of intensity based on their ability to lower LDL-C levels in the general population. At present, the debate between American and European guidelines is still open and, also considering the independent risk factor represented by HIV, specific guidelines for HIV-infected persons are warranted.

Another effective drug for the hypercholesterolemia is ezetimibe that markedly reduces the intestinal absorption of cholesterol. In HIV-infected subjects, ezetimibe already showed to be effective in reducing the total cholesterol and LDL-C levels when used alone or in combination with rosuvastatin. PCSK9 inhibitors, MTP inhibitors, antisense oligonucleotide against apolipoprotein B, adenosine Triphosphate Citrate Lyase Inhibitors are experimental and sometimes promising new classes of drugs for the treatment of hypercholesterolemia.

Fibrates represents the first choice for treatment of hypertriglyceridemia in HIV infected patients. Few studies evaluated the use of fibrates and statins in HIV associated dyslipidemia and demonstrated an higher efficacy as reported in the general population. Omega 3 fatty acids have a good safety profile and has been used in HIV-associated dyslipidemia, but their efficacy is limited to patients with a low-moderate dyslipidemia and isolated hypertrigliceridemia. Other drugs for hypertrigliceridemia are: acipimox, that showed a low efficacy; niacin that, in spite of its efficacy can induce insulin resistance and hepatotoxicity; and tesamorelin that has demonstrated a good efficacy in lowering triglycerides and total cholesterol.

## Abbreviations

- α: nuclear receptor peroxisome proliferator activator receptor-α
ABC: abacavir
ACC: American College of Cardiology
ACL: adenosine Triphosphate Citrate Lyase
AHA: American Heart Association
ART: antiretroviral therapies
ASCVD: atherosclerotic cardiovascular disease
ATV/r: atazanavir/ritonavir
cART: combination antiretroviral therapy
CCR5: C-C chemokine receptor type 5
CFA: circulating fatty acid
CK: creatine phosphokinase
CV: cardiovascular
CVD: cardiovascular disease
CYP: cytochrome P
D:A:D: Data Collection on Adverse Events of Anti-HIV Drugs study
EAS: European Atherosclerosis Society
EFV: efavirenz
EMA: European Medicines Agency
ESC: European Society of Cardiology
ETR: etravirine
EVG/COBI/FTC/TDF: elvitegravir/cobicistat/emtricitabine/tenofovir
FH: familial hypercholesterolemia
FPV/r: fosamprenavir/ritonavir
FTC-EFV-TDF: emtricitabine-efavirenz-tenofovir DF
FTC-RPV-TDF: emtricitabine-rilpivirine-tenofovir DF
HDL–C: high-density lipoprotein cholesterol
HMG-CoA: hydroxy-3-methylglutaryl coenzyme A
INIs: integrase inhibitors
LA: Lipoatrophy
LDL-C: low-density lipoprotein cholesterol
LDLR: LDL receptor
LH: Lipohypertrophy
LPV/r: lopinavir/ritonavir
mAbs: monoclonal antibodies
MI: myocardial infarction
mtDNA: mitochondrial DNA
MTP: microsomal triglyceride transport protein
NNRTI: non-nucleoside reverse-transcriptase inhibitors
NRTIs: nucleoside/nucleotide reverse transcriptase inhibitors
NVP: nevirapine
PI: protease Inhibitors
PLWHIV: people living with HIV infection
PRO: patient-Reported Outcomes
RAL: raltegravir
RPV: rilpivirine
RTV: ritonavir
STR: single-tablet regimen
TC: total cholesterol
TDF: tenofovir
TGL: tryglicerides
ULN: upper limit of normal
VLDL: very-low-density lipoproteins
